# Caregivers’ Experience with Lip Taping as a Presurgical Orthopedic Treatment for Cleft Lip and Palate Defects

**DOI:** 10.3390/children11030332

**Published:** 2024-03-10

**Authors:** Athar Thair, Mushriq Abid, Arkadiusz Dziedzic

**Affiliations:** 1 Orthodontic Department, College of Dentistry, University of Baghdad, Baghdad 10001, Iraq; nofous.thaer22031@codental.uobaghdad.edu.iq; 2 Department of Conservative Dentistry with Endodontics, Medical University of Siles, 40-055 Katowice, Poland; adziedzic@sum.edu.pl

**Keywords:** caregivers, cleft lip and palate, experience, survey

## Abstract

Background: This study aimed to assess caregivers’ experiences and perceptions of applying lip taping as presurgical orthopedic therapy (PSO) for infants with a cleft lip and palate. Methods: A retrospective survey was conducted, inviting parents to respond to a series of structured questions between September 2022 and June 2023. The questionnaire focused on evaluating parents’ experience with lip taping, a crucial component of PSO. Descriptive statistics and the Chi-square test were employed to analyze relationships between categorical variables. Results: Of the 157 participants, overall, 122 completed the survey, forming the dataset for analysis. A majority (90.0%) reported sufficient experience in using lip taping and no major difficulties with lip taping application. Caregivers with higher education levels exhibited a significantly positive correlation (*p* = 0.015). Additionally, 93.4% confirmed the efficacy of lip taping for premaxillary segment retraction, with this outcome being correlated with caregivers’ knowledge and education (*p* = 0.008). Interestingly, caregivers’ age also demonstrated a substantial association (*p =* 0.020). Conclusions: While a vast majority expressed positive experiences with lip taping as a presurgical treatment, continuous, tailored education on cleft lip and palate is imperative. This education should be directed towards caregivers and individuals offering direct support to parents of children with CLP, ensuring optimal care and preparation for surgical treatment.

## 1. Introduction

Cleft lip and palate (CLP) ranks among the most prevalent craniofacial anomalies affecting the head and neck [1], with a global incidence ranging from 1 to 7 per 1000 live births [2,3]. Constituting about 65% of non-tumor head and neck malformations, CLP poses challenges in feeding, hearing, speech, and psychological well-being [4,5]. The severity of the cleft can range from a small notch on the upper lip to a huge gap in the roof of the mouth, so this kind of malformation requires great attention and more understanding [6,7]. To address the multidimensional nature of CLP, a collaborative effort involving surgeons, orthodontists, and speech therapists is essential [8]. CLP patients also have esthetic and psychological problems, so it is preferable to manage them with the help of a team of specialists [7,9,10]. Of particular significance is the role of orthodontists, as efficient presurgical orthopedic management (PSO) before surgical lip closure is recognized as the foundational step in CLP treatment [11,12,13]. Initiated in the first days of life, PSO proves advantageous by enhancing feeding, reducing the cleft gap, and thereby improving the outcomes of subsequent palatal and lip repair procedures [14,15]. Prior to surgical lip closure, orthopedic treatment further aims to optimize maxillary–mandibular relationships for superior surgical outcomes [16].

Different surgical techniques are used for lip closure, but the ones most commonly used by surgeons are the Millard or Fisher technique for the repair of the unilateral cleft or the Millard technique for the repair of the bilateral cleft [17]. The main advantages of these surgical procedures are to complete the orbicularis oris muscle, gain symmetry, and, most importantly, provide cosmetics [18].

The historical progression of PSO includes Hoffmann’s introduction of a head cap in 1686, which applied retraction force to the premaxillary segment [19]. Advancements such as the use of silver wire to approximate alveolar segments were introduced [20]. In 1993, Grayson et al. introduced the Nasoalveolar Molding (NAM) concept, employing an intraoral plate with a nasal stent to guide growth and alignment in infants with CLP [21]. DynaCleft, a recent innovation, substitutes the intraoral plate with a nasal elevator and paper tape, simplifying the procedure [22].

Despite these advancements, a less explored yet promising technique is lip taping. Clinical assessments in a small number of trials, such as those by Dawjee et al. (2014) and Pool and Farnworth (1994), showed that it was useful in minimizing the cleft gap and molding maxillary alveolar segments [23]. Described as a simple and cost-effective approach, lip taping represents a valuable addition to the armamentarium of CLP treatment options [24]. As the field of CLP treatment evolves, continuous research and the introduction of new techniques, such as DynaCleft, signify ongoing progress and a commitment to enhancing patient outcomes [22].

The use of PSIOs is currently widely accepted [25,26] but there are disagreements over the long-term advantages of this kind of CLP therapy [27,28,29,30]. Nasoalveolar molding, lip taping, and other PSIO approaches offer the best treatment outcomes for presurgical newborn orthopedics, according to several systematic evaluations [11,31,32,33]. Diverse levels of awareness regarding cleft lip and palate (CLP) have been documented across rural and urban areas, highlighting regional disparities [34,35]. Importantly, parents’ education levels and knowledge about CLP are closely linked to the decision to forego treatment, thereby elevating the risk of adverse outcomes, including mortality in severe cases [35,36]. Furthermore, delays in surgical interventions and non-compliance with specialist instructions, such as adherence to presurgical orthopedic treatment, may result in unfavorable clinical outcomes, negatively impacting both the child and the family [37]. Given that presurgical orthopedics (PSO) is recommended as an initial therapeutic approach, with caregivers assuming a pivotal role in the care of children with CLP, lip taping is one of the simplest approaches for this treatment. Lip taping works to establish an acceptable maxillary–mandibular relationship, restore normal oral function, make surgical lip closure easier, and improve the general outcome [16]. This study aimed to quantitatively assess caregivers’ perceptions and experiences with the use of lip taping as an integral component of presurgical orthopedics. The limited available data on caregivers’ opinions about PSO underscore the pressing need for this current study, which seeks to bridge gaps in understanding and optimize caregiver involvement in the CLP treatment process. Caregivers of patients with cleft lip and palate from different areas of Iraq and from different medical centers that provide medical treatment for these patients were interviewed. The null hypothesis was evaluated comparatively using statistical tests.

## 2. Materials and Methods

### 2.1. Study Sample

**Figure 1:**
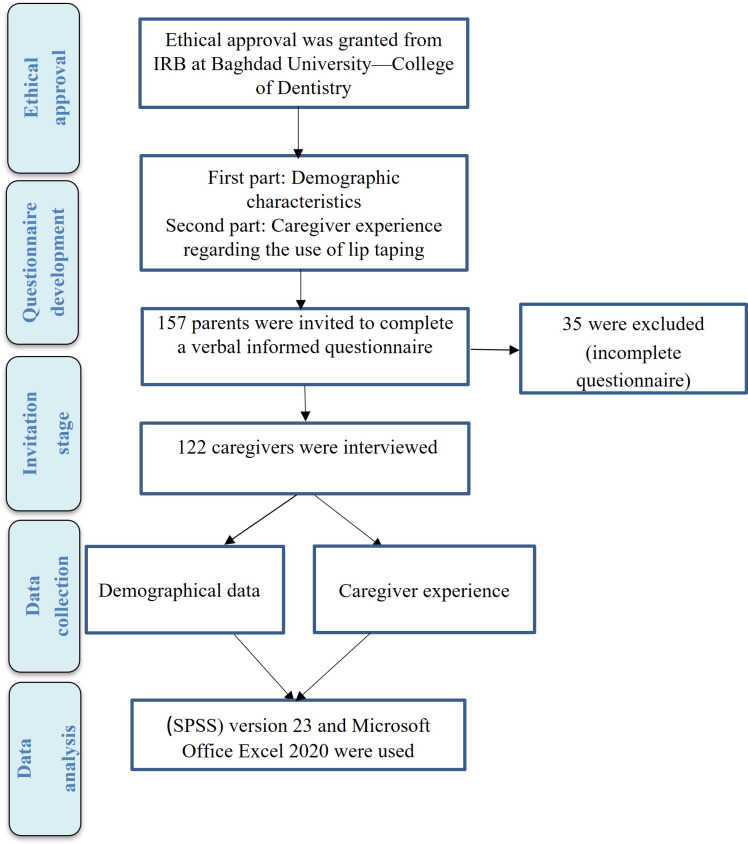
Study flowchart.

A retrospective survey was undertaken across three prominent clinical centers: the orthodontics department, the maxillofacial department, and the cleft center at the College of Dentistry at the University of Baghdad, where cleft lip and palate patients from different areas of Iraq are received to direct those patients’ families to the treatment protocol for their affected children, starting from the first day of infant life, with orthopedic treatment ending with the orthognathic surgical procedure that might be needed in the late teens. The primary study protocol is depicted in Figure 1 (flowchart). Inclusion criteria encompassed caregivers of both sexes, which could be parents or any other person from the family who was taking care of the affected infant, spanning all ages, caring for infants diagnosed with bilateral cleft lip and palate (CLP), and registered at the aforementioned clinical centers in Baghdad. The current survey was performed for caregivers of babies with bilateral cleft lip and palate who underwent orthopedic treatment by using lip taping prior to surgical lip closure to check their experience with such a type of orthopedic management.

### 2.2. Variables Recorded

A total of 157 questionnaires were distributed to eligible participants when they were interviewed, and data collection occurred between September 2022 and June 2023.

The authors constructed validated questionnaires that were orally administered and linguistically modified to Arabic. They comprised two sections: the initial segment, which aimed to gather essential demographic details such as caregivers’ age, education level, residency, and the presence of siblings affected with a cleft. The second part of the questionnaire comprised ten specifically crafted closed-ended questions to assess caregivers’ experiences with using lip taping as an orthopedic treatment for their infants. Q9 was explained by using a histogram, which shows the source of information about orthopedic treatment (whether it was a doctor, the media, or other families). Ethical approval was secured from the Institutional Review Board (IRB) at the College of Dentistry, University of Baghdad, on 12 January 2023 (reference number: 765, project 765423). The study adhered to the principles outlined in the World Medical Association Declaration of Helsinki. Participants provided written informed consent, and all identifiable data were meticulously anonymized to uphold participant confidentiality.

### 2.3. Sample Size

The determination of the study’s sample size was conducted by employing the following formula: [N = N/1 + Z2 × P (1 − P)/E2N], where N: population size, Z: z score for % confidence interval, E: margin of error, and P: population proportion (0.5). Based on a prior study indicating a moderate effect size of 0.3 [7], a study with 80% power necessitated a total sample size of 143 to assess caregivers’ experiences. This evaluation utilized a two-tailed test at a 5% level of significance. The verification of statistical power was executed using G*Power version 3.1.9.2.

### 2.4. Statistical Analysis

The data were analyzed using the statistical package for social science (SPSS) version 25 (SPSS Inc., Chicago, IL, USA). Descriptive statistics were used to define the characteristics of the study variables in the form of raw counts and percentages. A Chi-square test was used to assess relationships between categorical variables. A conventional *p*-value of less than 0.05 was the criterion for rejecting the null hypothesis.

## 3. Results

Out of 157 filled-out questionnaires, 122 surveys were eligible for data analysis, as they were fully completed, while 35 surveys were excluded to avoid bias due to their incompletion.

### 3.1. Reliability Test

Table 1 shows that the data are reliable, with an interclass correlation showing very good reliability (0.884), with confidence intervals ranging between 0.649 and 0.961.

### 3.2. Demographic Characteristics

Table 2 displays the demographic characteristics of the study participants. The survey revealed that nearly half of the caregivers fell within the 20–30 age range (n = 56, 45.9%). Conversely, the remaining half were aged over 30 (n = 56, 45.9%), with the remainder being under 20 years old. The majority of participants were found to be educated (n = 56, 45.9%), and a significant proportion resided in urban areas (n = 82, 67.2%). A small number reported having an affected sibling (n = 13, 89.3%).

### 3.3. Caregivers’ Experiences

**Figure 2:**
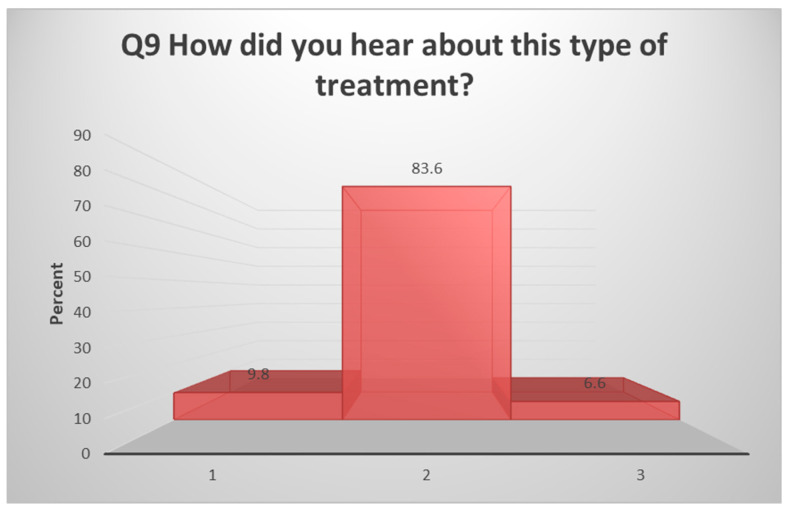
Knowledge about the PSO treatment: (**1**) refers to doctors, (**2**) refers to media, and (**3**) refers to the other parents.

All interviewed caregivers reported their babies undergoing orthopedic treatment at some point using lip taping. Table 3 illustrates the caregivers’ experiences with lip taping as an orthopedic treatment. The majority encountered no difficulty during tape application (90.0%), with only a small fraction facing challenges (9.0%). A significant portion (57.4%) reported no skin sensitivity in their babies, while 72.1% indicated that lip taping adequately covered the anomaly. Approximately three-quarters of caregivers (78.7%) noted no interference with baby feeding, and a majority expressed no embarrassment (74.3%) or safety concerns (83.6%) regarding lip taping, affirming its efficacy. Additionally, 93.4% of caregivers expressed satisfaction with the results. Complaints of tape slipping were reported by 27.0%, while 73.0% did not face such issues. Figure 2, represented in a histogram, emphasizes that over 80% of participants learned about this treatment through doctors, while less than 10% obtained information from other sources.

### 3.4. Relationship between the Caregivers’ Experiences and Their Education

Table 4 actively evaluates caregiver experiences with lip taping as a component of presurgical orthopedic treatment (PSO), categorizing them by caregiver education. Notably, 95.3% of educated caregivers found no difficulty in applying the tape, establishing a significant correlation (*p* = 0.015) between education and the ease of lip taping. Conversely, a considerable proportion of illiterate caregivers faced challenges during the lip-taping procedure. The experience of retracting the premaxilla was further substantially linked (*p* = 0.008) with education level, and 97.7% of educated caregivers attested to the effectiveness of lip tape in this regard. However, the remaining seven questions showed no significant relationship between caregiver education and their experience using lip taping.

### 3.5. Relationship between Caregivers’ Experiences with Using Lip Taping and Their Residency

In Table 5, a substantial 76.3% majority of caregivers residing in urban areas reported no slipping of the lip tape from its position during use, elucidating a significant correlation with a *p*-value of 0.031. Notably, the remaining questions displayed no significant relationship with the caregivers’ area of residency.

### 3.6. Relationship between Caregiver Experience with Using Lip Taping and Any Affected Siblings

Table 5 also showed that no relationship was found between the caregiver’s experience and having an affected sibling.

### 3.7. Relationship between Caregiver’s’ Experiences for Using Lip Taping as a Treatment and Their Age (n = 122)

With Table 6, we can see a significant correlation between caregivers age and their experience, with a p value of 0.017. The table reveals that all caregivers (100.0%) who are older than 30 years feel no embarrassment about having lip taping for their babies. On the other hand, those who were younger than 20 years, with a percentage of 83.3%, were shy about letting their babies wear the tape. The table also reveals a significant correlation (*p* = 0.020) between the efficiency of retracting the premaxillary segment by the tape and the age of the caregivers; most of those who were between the ages of 20 and 30 (98.2%) and those who were over 30 (92.6%) showed their satisfaction with the efficiency of the lip taping retraction effect. The rest of the questions showed no significant correlation.

## 4. Discussion

As we know, cleft lip and palate is a common congenital anomaly with an estimated incidence of 1–7 per 1000 births [3]. The failure of the fronto-nasal and maxillary processes to fuse leads to this anomaly, which can cause a cleft of variable severity that can penetrate the lip, alveolus, and nasal floor [38]. Such patients usually have several dental and medical problems, such as natal and neonatal teeth, microdontia, ectopically erupted teeth, supernumerary teeth, and congenitally missing teeth. In addition, they have many medical problems, like feeding problems, ear and chest infections, and speech difficulties that require directing the family to overcome them [39,40].

The first line for management of cleft lip and palate in infants is to start with an orthopedic treatment in the first days of their lives. Despite the benefits of PSIOs, great controversy still exists globally related to the orthopedic management of infants with CLP before surgery [41]. Both caregivers and clinicians find it challenging to deal with infants with CLP during their first days of life [42]. Since surgical correction is considered the ultimate goal to correct the anatomical defect, it is sometimes difficult to provide this surgical correction during the early months of an infant’s life. As a result, the family usually faces considerable adverse functional and psychological problems that require medical intervention. That is why PSIOs are thought to reduce these problems and provide a smooth transitory period before surgery [43].

Given the limited evidence on caregivers’ experiences with cleft lip and palate (CLP) management, this retrospective study aimed to assess caregivers’ experiences with using lip taping as a presurgical orthopedic treatment. The hypothesis sought to examine this experience in relation to caregivers’ education, area of residency, age, and whether they had affected siblings.

The study revealed varying caregiver experiences with lip taping, emphasizing the impact of the area of residency on awareness levels regarding CLP defects. Different levels of awareness were noted in urban and rural areas [34,35]. Additionally, caregivers’ education emerged as a crucial factor, with low education levels correlating closely with a tendency to leave children untreated [35,36].

The results indicated that 90.0% of caregivers faced no difficulties with lip taping, with a significant correlation observed among the educated participants. Moreover, 93.4% acknowledged the efficacy of lip taping in retracting the premaxillary segment, a percentage significantly associated with caregivers’ education. This underscores the pivotal role of education in ensuring optimal care for infants with CLP. Age-related findings indicated a significant correlation between caregiver age, efficacy in retracting the premaxillary segment, and the absence of embarrassment among those above 30 years. This emphasizes the importance of caregiver age in delivering desirable care for infants with CLP. A noteworthy correlation was observed between area of residency and caregivers’ attitudes, with 76.3% residing in urban areas. These findings align with previous research highlighting the impact of the residency on parental and caregiver awareness and experience [34,35]. Interestingly, no significant relationship was found between caregiver experience and having affected siblings, possibly influenced by the limited number of participants with affected siblings, hindering a robust comparison.

Magyar and his colleagues conducted a survey-based study in 2022 that confirmed the findings of this study [44]. The study utilized a 32-item questionnaire following NAM therapy and involved 17 families. The study showed a good parental experience and satisfaction with the results of the therapy, and they would advise other caregivers to use it with CLP patients.

Another study carried out in 2016 by Hopkins and his colleagues [45] explored the experiences of eight mothers and four fathers who were interviewed and were taking care of infants with CLP who were receiving molding. They were keen on the treatment process and revealed the benefits of such treatment, which supports our findings. In addition to the previous studies, in 2016, another study was carried out by 53 participating CLP caregivers whose infants were receiving treatment by NAM. Most of those caregivers stated that the molding technique made a great change in the patient’s lip (50 caregivers, 94.3%), palate (48 caregivers, 90.5%), and nose (50 caregivers, 94.3%), and 52 caregivers (98.1%) reported that the defect was improved, which revealed caregivers’ experiences [46].

In conclusion, the management of children with CLP necessitates a multidisciplinary team, especially in severe cases. All medical and dental professionals share the responsibility of educating caregivers of patients with CLP about the significance of early orthopedic treatment and its role in optimizing future outcomes.

### 4.1. Strengths and Limitations of the Study

The utilization of a multicenter study design enhances the reliability and robustness of the data collected within regional areas. However, owing to the retrospective nature of the data collection, there is a potential for imprecise information. Therefore, to overcome these limitations, future research endeavors may consider implementing a cross-sectional or prospective study design.

### 4.2. Implications

The results gained could have a significant impact on public health programs that target CLP patients. Additionally, they can offer evidence-based data to support policy decisions made by authorities on the healthcare management of CLP cases. Educating people about the importance of PSIOs in different areas by using different means and including all age groups could improve the treatment results for this type of patient.

## 5. Conclusions

The findings of this study highlight a positive inclination among caregivers of children with cleft lip and palate towards the lip-taping procedure, who consider it an integral component of presurgical orthopedics. Interestingly, a noteworthy correlation was observed between specific caregiver characteristics—including education level, age, and area of residency—and the acceptance of initiating PSO during the early stages of a child’s life with CLP.

## Figures and Tables

**Table 1:** Reliability test.

Interclass Correlation		95% Confidence Interval	
		Lower Bound	Upper Bound
Average Measures	0.884	0.649	0.961

**Table 2:** Characteristics of the study participants.

Category	n	%
**Age**		
<20 years	12	9.80%
20–30 years old	56	45.90%
>30 years	56	45.90%
**Level of education**		
Educated	56	45.90%
Illiterate	36	29.50%
**Area of residency**		
Urban	82	67.20%
Rural	40	32.80%
**Any affected siblings**		
Yes	13	10.70%
No	109	89.30%

n: number of participants.

**Table 3:** Caregiver experience toward lip taping as a presurgical orthopedic treatment for infants with cleft lip and palate.

Questions	Yes	n%	No	n%
Q1: Did you face any difficulty when applying the tape?	11	9.0%	111	90.0%
Q2: Did the tape cause any irritation or sensitivity to the child cheek?	50	42.6%	70	57.4%
Q3: Did the tape cover the anomaly properly?	88	72.1%	34	27.9%
Q4: Did the tape interfere with the baby feeding, crying or smiling?	26	21.3%	96	78.7%
Q5: Did the tape frequently peel off?	57	46.7%	65	53.3%
Q6: Did you face any embarrassment that your baby wearing a tape?	7	5.7%	115	74.3%
Q7: Did the elastic or tape cause any injury to the baby?	20	16.4%	102	83.6%
Q8: Did the tape work efficiently and retract the premaxilla?	114	93.4%	8	6.6%
Q10: Did the tape slip off its position?	33	27.0%	89	73.0%

n: number of participants.

**Table 4:** The association between caregivers’ experiences with using lip taping as a treatment and their education (n = 122).

Questions	Educated			Illiterate		Statistical Analysis	
		n = 56	45.9%	n = 36	29.5%	X^2^ (df = 2)	*p*
Q1: Did you face any difficulty when applying the tape?	Yes	4	4.7%	7	19.4%	6.7	0.015 *
	No	82	95.3%	2	80.6%		
Q2: Did the tape cause any irritation or sensitivity to the child cheek?	Yes	20	55.6%	32	37.2%	3.493	0.048 *
	No	16	44.4%	54	62.8%		
Q3: Did the tape cover the anomaly properly?	Yes	15	17.4%	11	30.6%	2.60	0.145
	No	25	69.4%	71	82.6%		
Q4: Did the tape interfere with the baby feeding, crying or smiling?	Yes	41	47.7%	16	44.4%	0.106	0.843
	No	45	52.3%	20	5.6%		
Q5: Did the tape frequently peel off?	Yes	4	4.7%	3	8.3%	0.636	0.420
	No	82	95.3%	33	91.7%		
Q6: Did you face any embarrassment that your baby wearing a tape?	Yes	13	15.1%	7	19.4%	0.347	0.596
	No	73	84.9%	29	80.6%		
Q7: Did the elastic or tape cause any injury to the baby?	Yes	84	97.9%	30	83.3%	8.518	0.008 *
	No	2	2.3%	6	16.7%		
Q8: Did the tape work efficiently and retract the premaxilla?	Yes	84	97.9%	30	83.3%	8.518	0.008 *
	No	2	2.3%	6	16.7%		
Q9: How did you hear about this type of treatment?	Media	10	11.6%	2	5.6%	2.461	0.337
	Doctor	69	80.2%	33	91.7%		
	Other	7	8.1%	1	2.8%		
Q10: Did the tape slip off its position?	Yes	20	23%	23	63.9%	2.125	0.181
	No	66	76.7%				

df: degrees of freedom; *p*: level of significance; *: statically significant.

**Table 5:** The associations between caregivers’ experiences with using lip taping as a treatment and their residency and having any affected siblings (n = 122).

Questions	Rural			Urban		Statistical Analysis		Affected Siblings		No Affected Siblings		Statistical Analysis	
		n = 40	32.8%	n = 82	67.2%	X^2^ (df = 2)	*p*	n = 13	10.0%	n = 109	89.3%	X^2^ (df = 2)	*p*
Q1: Did you face any difficulty when applying the tape?	Yes	6	15.0%	5	6.1%	2.597	0.174	1	7.7%	10	9.2%	0.031	1.000
	No	34	85.0%	77	93.9%			12	92.3%	99	90.8%		
Q2: Did the tape cause any irritation or sensitivity to the child cheek?	Yes	17	42.5%	35	42.7%	0.000	1.00	4	30.8%	48	44.0%	836	0.395
	No	23	57.5%	47	57.3%			9	69.2%	61	56.0%		
Q3: Did the tape cover the anomaly properly?	Yes	28	0.0%	60	73.2%	0.134	0.830	11	84.6%	77	70.6%	1.128	0.513
	No	2	30.0%	22	26.8%			2	15.4%	32	29.4%		
Q4: Did the tape interfere with the baby feeding, crying or smiling?	Yes	11	27.5%	15	18.3%	1.359	0.346	0	0.0%	26	23.9%	3.941	0.068
	No	29	2.5%	67	81.7%			13	100.0%	83	76.1%		
Q5: Did the tape frequently peel off?	Yes	21	52.5%	36	43.9%	0.798	0.441	7	53.8%	50	45.9%	0.297	0.770
	No	19	47.5%	46	56.1%			6	46.2%	59	54.1%		
Q6: Did you face any embarrassment that your baby wearing a tape?	Yes	5	12.5%	2	2.4%	0.636	0.420	0	0.0%	7	6.4%	0.886	1.00
	No	35	87.5%	80	97.6%			13	100.0%	102	93.6%		
Q7: Did the elastic or tape cause any injury to the baby?	Yes	4	10.0%	16	9.5%	1.775	0.206	2	15.4%	18	16.5%	0.11	1.000
	No	36	90.0%	66	80.5%			11	84.6%	91	83.5%		
Q8: Did the tape work efficiently and retract the premaxilla?	Yes	36	90.0%	78	95.1%	1.151	0.436	13	100.0%	101	92.7%	1.021	0.598
	No	4	10.0%	4	4.9%			0	0.0%	8	7.3%		
Q9: How did you hear about this type of treatment?	Media	3	7.5%	9	11.0%	2.461	0.337	0	0.0%	12	11%	2.853	0.389
	doctor	36	90.0%	66	80.5%			13	100.0%	89	81.7%		
	other	1	2.5%	7	8.5%			0	0.0%	8	7.3%		
Q10: Did the tape slip off its position?	Yes	16	40.0%	17	20.7%	5.58	0.031 *	4	30.8%	29	26.6%	0.102	0.747
	No	24	60%	65	76.3%			9	69.6%	80	73.4%		

n: number of participants; *: statistically significant.

**Table 6:** The association between caregivers’ experiences with using lip taping as a treatment and participant age (n = 122).

Questions	<20			20–30		>30		Statistical Analysis	
		n = 12	9.8%	n = 56	45.9%	n = 56	45.9%	X^2^ (df = 2)	*p*
Q1: Did you face any difficulty when applying the tape?	Yes	1	8.3%	5	8.9%	5	8.9%	0.011	1.000
	No	11	91.7%	51	91.1%	51	91.1%		
Q2: Did the tape cause any irritation or sensitivity to the child cheek?	Yes	4	3.3%	28	50.0%	20	37%	2.359	0.293
	No	8	6.7%	28	50.0%	34	63%		
Q3: Did the tape cover the anomaly properly?	Yes	9	75.0%	41	73.2%	38	70.4%	0.165	0.956
	No	3	5.0%	15	26.8%	16	29.6%		
Q4: Did the tape interfere with the baby feeding, crying or smiling?	Yes	4	3.3%	9	16.1%	13	24.1%	2.197	0.335
	No	8	6.7%	47	83.9%	41	75.9%		
Q5: Did the tape frequently peel off?	Yes	7	8.3%	22	39.3%	28	51.9%	2.465	0.303
	No	5	41.7%	34	60.7%	6	48.1%		
Q6: Did you face any embarrassment that your baby wearing a tape?	Yes	2	16.7%	5	8.9%	0	0.0%	0.991	0.017 *
	No	10	83.3%	51	91.1%	54	100.0%		
Q7: Did the elastic or tape cause any injury to the baby?	Yes	2	16.7%	10	17.9%	8	14.8%	0.186	0.937
	No	10	83.3%	46	82.1%	46	85.2%		
Q8: Did the tape work efficiently and retract the premaxilla?	Yes	9	75.0%	55	98.2%	50	92.6%	8.806	0.020 *
	No	3	25.0%	1	1.8%	4	7.4%		
Q9: How did you hear about this type of treatment?	Media	0	0.0%	7	12.5%	5	9.3%	3.543	0.655
	Doctor	12	100%	46	82.1%	44	81.5%		
	Other	0	0.0%	3	5.4%	5	9.3%		
Q10: Did the tape slip off its position?	Yes	5	41%	14	25.0%	14	25.9%	2.125	0.181
	No	7	58.3%	42	75.0%	40	74.1%		

*: statically significant.

## Data Availability

The data will be available on request from the corresponding author. The data are not publicly available due to privacy.
